# Supplementary material for: A feasibility study of behavioural activation for haemodialysis: The BEACH study protocol
Source: PLoS One. 2026 Aug 6;21(8):e0353407. doi: 10.1371/journal.pone.0353407 (PMC13446654; doi:10.1371/journal.pone.0353407)
Supplement: Supplementary file 1 [file pone.0353407.s001.docx]

The BEACH Study: BEhavioural ACtivation on Haemodialysis

Feasibility Study Protocol

**Protocol version no.: v1.3	Date: 08.01.2026**

**Grant reference number: NIHR209283**

**IRAS reference number: 361821**

**ISRCTN: ISRCTN74685186**

| **Document History** | | | |
| --- | --- | --- | --- |
| **Version** | **Date** | **Editor** | **Comments** |
| 1.0 | 08/08/2025 | Claire Carswell, Sunil Bhandari, Kate Bramham, Joe Chilcot, Lina Gega, Fez Awan | Initial drafting and review comments |
| 1.1 | 17/09/2025 | Claire Carswell, Sunil Bhandari, Kate Bramham, Joe Chilcot | Review comments |
| 1.2 | 25/09/2025 | Claire Carswell | Update details on the screening process |
| 1.3 | 08/01/2026 | Claire Carswell | Added end-of-study definition |

Contents

**Introduction	3**

Aims and objectives	5

**Materials and Methods	5**

Study Design	5

Research setting	5

Feasibility Cluster Randomised Control Trial	6

Study Population	6

Sample size	7

Recruitment	7

Randomisation	8

Intervention	9

Intervention fidelity	10

Usual Care	11

Data collection	11

Baseline demographic and clinical data	11

Feasibility measures	11

Clinical Outcome Measures	12

Data management and storage	12

Data Analysis	13

Feasibility Economic Evaluation	13

Data collection	13

Data Analysis	14

Data management and storage	14

Process evaluation	14

Participants	14

Recruitment	15

Data collection	15

Data Analysis	16

Data management and storage	16

Progression Criteria	16

Ethical considerations	17

Informed consent	17

Confidentiality and anonymity	17

Welfare of participants	18

Welfare of the research staff and facilitators	19

# Introduction

People who develop kidney failure are required to undergo relentless and challenging treatment to sustain life. While the gold-standard treatment for kidney failure is kidney transplantation, many people are not suitable for transplantation, are unable to find a donor, or must wait until a donor is found. In these situations, people typically rely on haemodialysis, an invasive treatment that requires them to attend the hospital usually three times a week for four hours each time, during which they are restricted to a bed or chair, and may experience troubling side effects such as nausea, cramping, pruritus and significant fatigue(1). This can have a profound impact on a person’s mental health and well-being, resulting in significantly high rates of depression and anxiety. Depending on the method of assessment, depression rates amongst people receiving dialysis range from 22.8% to 39.3%(2) while the prevalence of depression in the UK general population is approximately 7.5%(3).

Depression has serious implications for the overall health of people receiving haemodialysis. Depression is associated with decreased quality of life and increased morbidity(4) and is an independent predictor of mortality, in part due to the impact that depression has on adherence to treatment(4). Treatment regimens involve not only haemodialysis, but strict fluid and dietary restrictions, alongside medication(1). People experiencing depression are less likely to be adherent to their treatment regimen(4), increasing their risk of hospitalisation, morbidity and death(5). Therefore, the issue of depression must be addressed amongst people receiving haemodialysis, to improve long-term outcomes for patients, their families and carers and reduce the economic burden on the health service.

Improving mental health support for people receiving haemodialysis has been identified as a priority by Kidney Care UK’s Psychosocial Health – A Manifesto for Action (6). This manifesto highlights the rising demand for better mental health support for people with kidney disease, occurring in parallel to declining resources. This lack of support is further exacerbated by the lack of screening for depression and the lack of evidence-based interventions to prevent or treat depression amongst people receiving haemodialysis (7). There is limited evidence on the effectiveness of antidepressant medication amongst people receiving haemodialysis (8), in part because of the difficulties in trial design, the adverse events associated with medication (9), and issues around feasibility, such as the existing treatment burden and prioritisation of kidney disease (10).

Cognitive behavioural therapy (CBT), another evidence-based treatment for depression, appears to be more effective at reducing symptoms of depression among people with kidney disease(11). However, issues surrounding long-term implementation in practice, such as the need for a trained clinical psychologist to deliver the intervention (12), the requirement to attend separate appointments (either in-person, online, or over the phone) (13), raise concerns about feasibility when resources are significantly limited and patients already face a substantial treatment burden (8). Therefore, novel evidence-based techniques to address depression in people receiving haemodialysis are needed, which can be delivered in a way that is acceptable and feasible.

Behavioural activation (BA) is an evidence-based psychological therapy that forms a key part of CBT. BA is based on making small changes in daily life to disrupt negative cycles of thoughts, feelings, and behaviour (14). People set their own pace and priorities, identifying activities that they personally find enjoyable to engage in. While there is evidence that CBT can improve depression among people with chronic kidney disease (CKD), there is a lack of evidence supporting the effectiveness of BA alone (11); however, it has been shown to be effective in the treatment and prevention of depression in the general population (15), and can be more cost-effective than traditional CBT(16). BA has also been shown to be effective in treating depression amongst people with long-term conditions in the short and medium term, while longer-term effects are being established (14, 17). Consequently, BA is recommended by the National Institute for Health and Care Excellence (NICE) guidelines on the management and recognition of depression in adults with chronic physical health problems(18).

There is a growing recognition of the need to integrate mental and physical health services, particularly to benefit people who have long-term physical health conditions, such as kidney failure (19-21). This integration is of particular importance for people receiving haemodialysis, due to the impact of the treatment on mental health (22, 23). A specific benefit of BA for this population is that it can be delivered outside of mental health services and has been successfully integrated into other services, such as primary care, where it has been delivered by trained non-specialists(24-27). A key issue in the feasibility of many mental health interventions for people receiving haemodialysis is the substantial treatment burden associated with haemodialysis, making additional appointments or the involvement of new healthcare professionals less acceptable (28). Therefore, BA that is delivered within the haemodialysis setting could provide an acceptable and culturally congruous method for treating depression in this patient group, particularly where resources for mental health support are limited.

However, while there is a need to establish the effectiveness of BA for reducing symptoms of depression among people receiving haemodialysis, the feasibility of integrated BA in haemodialysis settings is unknown(11). Therefore, a feasibility study is needed to determine whether the integrated delivery of BA is possible and acceptable before any assessment of effectiveness (29, 30). The proposed study will inform the development of a protocol for a definitive trial of an integrated BA intervention for people receiving haemodialysis and explore the acceptability and feasibility of screening for depression in this patient population(13). Finally, we will conduct workshops to develop a set of recommendations for future practice, policy, and research on managing depression among long-term conditions with high treatment burdens, based on the findings from the feasibility study.

## Aims and objectives

This study aims to establish the feasibility and acceptability of a behavioural activation intervention, integrated into haemodialysis care, to address symptoms of depression and anxiety amongst people with kidney failure who are receiving maintenance haemodialysis. To achieve this, we will meet the following objectives:

- Determine the feasibility of recruiting, randomising, and retaining participants, including screening for depression using the PHQ-4, in a definitive cRCT.
- Evaluate the feasibility of data collection methods within a cRCT.
- Evaluate the feasibility of conducting a future economic evaluation of an integrated behavioural activation intervention.
- Explore the acceptability of the integrated behavioural activation intervention for patients, healthcare professionals and informal carers.
- Refine the process for evaluating intervention fidelity in a definitive cRCT.
- Explore the generalisability of findings to other long-term conditions in knowledge transfer workshops.

# Materials and Methods

### Study Design

This study comprises a comprehensive mixed-methods feasibility study with three main components: a feasibility cluster randomised controlled trial (cRCT), a feasibility economic evaluation, and a process evaluation. This protocol is reported in accordance with the SPIRIT checklist for trial protocols.

### Research setting

This study will take place in two haemodialysis units, one in Hull and one in London. The Hull haemodialysis unit provides treatment to approximately 344 patients undergoing maintenance haemodialysis, while King’s College Hospital provides treatment to approximately 720 patients undergoing maintenance haemodialysis. Patients who receive haemodialysis typically attend three, four-hour-long sessions each week. At units in the UK, haemodialysis patients have set ‘shift patterns’ that they attend each week, either every Monday, Wednesday, and Friday, or every Tuesday, Thursday, and Saturday, and they attend at the same time on those days, either in the morning, afternoon, or evening (1). Patients at both King’s College Hospital and Hull are evenly split across these shift patterns.

Recruiting from two different geographic areas with varying levels of deprivation will enable us to explore different clinical and socioeconomic factors that may influence recruitment and participation in a definitive trial. SB is a consultant nephrologist at Hull University Teaching Hospitals NHS Trust, and KB is a consultant nephrologist at King's College Hospital NHS Foundation Trust.

## Feasibility Cluster Randomised Control Trial

### Study Population

**Inclusion criteria:**

Patients will be eligible for participation if they:

- Are over the age of 18 years.
- Are receiving maintenance in centre haemodialysis for > 3 months
- Have the capacity to provide informed consent.
- Score ≥ 3 on the PHQ-4

Depression is not regularly screened for within practice, despite recommendations from recent reports on depression in people with kidney disease (6). There is also evidence that patients receiving haemodialysis are reluctant to undergo screening for depression (31). Therefore, we will be unable to screen using routinely collected data. Instead, we will screen all potentially eligible patients at each unit using the PHQ-4. This is a valid ultra-brief tool that can detect both anxiety and depression. Patients who score three or higher on the PHQ-4 will be invited to participate in the study (32).

We will collaborate with our PPI panel to develop strategies that enhance the acceptability of screening for the feasibility study. We will then capture the proportion of people who are willing to be screened using the PHQ-4 and explore the barriers and facilitators of using the PHQ-9 as a screening tool within this population during the qualitative process evaluation.

We have kept the inclusion criteria broad in relation to depressive categories to explore the feasibility of delivery across different levels of severity and identify the proportion of people within each category. Low-intensity psychological interventions, such as BA, are recommended for people with long-term conditions and sub-clinical, mild to moderate depression (18). While high-intensity psychological interventions are recommended for people with long-term conditions and severe depression, BA is still also recommended (18).

We will also include people who are established on antidepressant treatments or have been attending talking therapy long-term (for over three months), through either their GP or mental health service, assuming they are still screening positive on the PHQ-4. We will record the proportion of patients eligible who are already established on treatment at the start of the trial, as well as any changes to their current care during the study, including changes in dose, type of medication, discontinuation, or cessation of psychosocial support or therapy. Participants who are eligible but already established on antidepressant treatment or longer-term psychological therapy may not be responding to these treatments, or these treatments may be ineffective. Therefore, these participants may represent a distinct subgroup in the definitive trial that may need to be assessed separately.

**Exclusion criteria:**

Patients will be ineligible for participation if they:

- Started antidepressant medication or forms of talking therapy within the previous three months.
- Are receiving haemodialysis for acute kidney injury
- Have a planned kidney transplant within the study period
- Are planning to move from the dialysis unit during the study period, either because they are moving home to a different catchment area, are planning to move to a satellite dialysis unit, or are planning to start home haemodialysis
- Do not have the capacity to provide informed consent. The clinical judgement of the dialysis team will determine the capacity to consent. We will develop recruitment materials for participants who may require additional communication support, in line with ASSENT guidance, to ensure we are not unfairly excluding eligible patients.
- Are unable to understand and communicate effectively in the English language.

### Sample size

As this is a feasibility study, a power calculation was not conducted, as we are not looking to measure

effectiveness. There is no consensus on the appropriate sample size for a feasibility study (33, 34). However, a total sample size of 52 people would allow us to estimate the recruitment rate with a precision of ±9 % (assuming a 40% recruitment rate previously observed and a 95% confidence interval). This provides an appropriate level of precision to inform the likely number of people the full-scale trial might need to screen. Given that we have four clusters, this would also allow for a minimum of 12 participants per cluster, with a total of 26 participants recruited from each site (35).

This will also enable us to have an adequate and diverse pool of participants for the qualitative exploration of feasibility and acceptability (36), ensuring that modifications to screening, recruitment, trial design, or intervention design and delivery can be identified in preparation for a definitive trial.

### Recruitment

Potentially eligible patients will be identified by healthcare workers at each site to ensure they have the capacity to consent to research. These patients will be approached to undergo screening using the PHQ-4 questionnaire. Kidney Care UK’s psychosocial manifesto recommends annual screening for psychosocial care needs (6). Still, depression screening is not currently part of regular care in haemodialysis units in the UK. There are concerns about the acceptability of screening tools among people receiving haemodialysis(28). In previous feasibility studies, 44% of haemodialysis patients consented to screening with the PHQ-9 (28). Based on the current patient numbers between the two sites, we estimate that approximately 466 patients would consent to screening (316 at KCH and 150 at Hull). Cross-sectional studies have shown that 53.7% of patients receiving haemodialysis in the UK have mild-severe depression when screened with the PHQ-9 (31). Assuming this trend holds for patients who consent to screening, we’d expect to identify approximately 169 eligible patients at KCH and 80 at Hull.

Patients who score three or above on the PHQ-4 will be invited to participate. All patients who screen positive using this threshold will be provided with information on support services already available to them through the renal unit or third-sector organisations, such as Kidney Care UK. We will develop risk management protocols for patients who meet the criteria for severe depression or who are identified as at risk for self-harm or suicide during baseline data collection. Our PPI panel will review all participant-facing documentation to ensure the language is appropriate and that the information is presented in an accessible manner. Screening logs will be used to capture the proportion of patients who are eligible for screening, the proportion of people who are eligible following screening, and the proportion of participants who consent and are randomised.

### Randomisation

Following the collection of baseline data from all enrolled participants, patients will be grouped into clusters based on their dialysis shift patterns. These shift patterns will be randomly assigned to either the control group, where enrolled participants will receive enhanced usual care, or the intervention group, where enrolled participants will receive the intervention. We will explore the feasibility of cluster randomisation according to the dialysis shift pattern.

The intervention will be delivered in the haemodialysis unit during treatment; therefore, individual randomisation is likely to result in control group participants observing the delivery of the intervention, resulting in contamination. Cluster randomisation will be used to assess whether this method is a feasible approach to reduce the risk of contamination. Patients attend the hospital for haemodialysis three times a week in a set shift pattern that determines the day of the week and the time they attend. Patients either attend haemodialysis in the morning, afternoon, or evening, and will either attend on a Monday, Wednesday, Friday, or Tuesday, Thursday, Saturday pattern (1).

While patients rarely change these pre-set dialysis shift patterns, we will record any instances where shifts are changed, and instances of actual or potential contamination during intervention delivery to determine the feasibility of cluster randomisation. This will include documenting instances when control participants observe the intervention being delivered, as well as the number of times participants had to change their shift to a different day (regardless of whether an intervention session is observed). During the feasibility study, we will collaborate with our PPI panel to explore methods for reducing potential instances of contamination or mitigating their impact if they occur. Additionally, we will investigate any differences in demographics or clinical characteristics across the different shift patterns and clusters, to inform randomisation in a future definitive RCT. We will also explore the feasibility of this form of randomisation in the qualitative process evaluation through interviews with both control and intervention participants, as well as with healthcare workers in the unit.

Figure 1. Proposed dialysis shift clusters at each site for randomisation.

### Intervention

A BA workbook was adapted from the ComBAT intervention (37) and other BA interventions specifically designed for individuals with long-term conditions. This workbook was then shared with the PPI panel for feedback and identification for further adaptation.

The integrated BA intervention will be delivered over a period of 6-8 weeks. Each participant allocated to the intervention group will receive a minimum of 6 one-to-one sessions, delivered at their bedside during their haemodialysis treatment. Participants will receive a BA workbook to support the content of each one-to-one session and to refer to between sessions.

At the end of each session, the facilitator and participant will plan take-away activities to do outside of the sessions. In each follow-up session, the participant will begin by checking in with the facilitator and reviewing their progress from the previous week.

Figure 2. Overview of BEACH intervention

Using a standardised training programme, we will train kidney health professionals to deliver the BA intervention as facilitators. The training will be delivered in one face-to-face workshop. The workshop includes both a theoretical component to explain what BA is and how it works, as well as a skills-based component featuring case scenarios and role-plays. In preparation for the training, the healthcare workers will complete a 3-hour Massive Open Online Course (MOOC) on BA, authored by LG, which is freely available on the international platform FutureLearn. (38) As part of the training, the intervention facilitators will be encouraged to adopt a person-centred and flexible approach to intervention delivery. While the content will be organised into 6 core sessions, the pacing of session content will be tailored to the participants’ needs. We will use a train-the-trainer approach when recruiting further facilitators, with initial sessions supervised by the initially trained BA facilitators. Following the training, the BA facilitators will be offered supervision by LG as needed, both individually and in groups, at most once a week for 30 minutes and at least once a month for 1 hour. This model has been successfully applied for the training and supervision of non-mental health professionals to deliver BA to clinical populations (39) .

We will assess the acceptability of the training through a qualitative process evaluation to inform any adaptations to the training in a definitive trial.

#### Intervention fidelity

Intervention fidelity will be evaluated using session logs and the bespoke “Aspects of Care Questionnaire”. The BA facilitators will be asked to complete a session log following each session, which will record session length and contain a menu of different intervention components (e.g. activity scheduling, psychoeducation, general counselling) that could be delivered during the session. These will be reviewed to identify whether core aspects of BA have been delivered as intended.

At follow-up, all participants will be invited to complete the Aspects of Care Questionnaire to help assess fidelity and contamination. The questionnaire has eight items that correspond to general activities that may have taken place within both randomisation arms (e.g. “I had an opportunity to talk about my current problems/general life”) as well as BA-specific activities (e.g, “I wrote down things I did for pleasure and necessary tasks on a weekly calendar”). We will compare the responses between the BA and control group participants to determine whether BA participants received core components of the intervention as intended (fidelity) and whether control participants inadvertently received BA components (contamination)(37).

### Usual Care

There is no standardised usual care for depression provided within haemodialysis units across the UK, and few units implement NICE guidelines for the assessment and management of depression in long-term conditions. Usual care typically takes the form of either internal or external signposting to services, either within the NHS, primary care or through third-sector providers such as Kidney Care UK. Therefore, the control group participants will be provided with information on available services and sign-posted to the relevant service if they wish to receive additional support (for example, the counselling service provided by Kidney Care UK).

### Data collection

#### Baseline demographic and clinical data

We will collect data on participants’ age, sex, ethnicity, employment status, Index of Multiple Deprivation, time on dialysis, primary renal diagnosis, and comorbidities. We will also collect self-reported data on whether participants have received a diagnosis of depression at any time in their life, when, if they are receiving any treatment for mental health conditions, and what that treatment entails.

#### Feasibility measures

The main outcome of the pilot cRCT is feasibility, which will be evaluated through the collection of descriptive data on recruitment, retention, and participants’ willingness to be randomised, in accordance with the CONSORT guidelines for feasibility and pilot trials (40). The following data will be collected:

- **Willingness to screen:** The proportion of individuals who agree to undergo the initial screening for depression with the PHQ-4.
- **Eligibility Criteria Suitability:** The number of individuals excluded from the trial and the specific reasons for exclusion will be recorded to assess the appropriateness and practicality of the inclusion and exclusion criteria. Reasons for ineligibility and non-participation will be collected in the screening logs.
- **Willingness to Participate/ Recruitment rate:** The proportion of eligible individuals who consent to participate in the trial will be recorded as a measure of feasibility and acceptability. If significant barriers arise consistently during the initial recruitment period, we will consult with members of our PPI panel to develop strategies to mitigate these barriers. We aim to recruit 52 participants from two sites within three months, based on recruitment rates from other feasibility studies of intradialytic interventions (41).
- **Retention Rates:** The proportion of randomised participants who remain in the study and complete the follow-up assessments will be documented. Follow-up contact will be made, where possible, to ascertain reasons for discontinuation in cases where participants have dropped out.
- **Intervention adherence:** The proportion of participants allocated to the intervention group who complete the intervention, as well as the proportion of missed and incomplete sessions, will be recorded in session logs.
- **Time required for data collection and analysis:** Time for screening, consent, and follow-up will be recorded via timesheets to inform timelines and milestones in a future trial.
- **Feasibility of outcome measures**: The Proportion of missing data from each of the outcome measures will be reported to determine the feasibility of collecting complete data using the identified outcome measures.

These measures of feasibility and acceptability will also be explored in more detail in the qualitative process evaluation.

#### Clinical Outcome Measures

As the focus of this study is on the feasibility and acceptability of the BA intervention, we will not evaluate its effectiveness (42). Instead, we will explore the feasibility of collecting clinical outcome data and the acceptability of the outcome measures for use within a future cRCT.

Most studies evaluating BA have a follow-up period under three months (17); therefore, we will administer the clinical outcome measures at baseline and 3 months post-randomisation. The clinical outcomes will include depression, as measured by the PHQ-9, a commonly used measure of depression that has been psychometrically validated within people who have kidney failure. (43) We will also capture anxiety, measured using the GAD-7, health-related quality of life, measured by the KDQOL-SF36 (44, 45), mental well-being, measured by the WEMWBS (46), and adherence to haemodialysis, measured by dialysis attendance captured in the healthcare resource use logs, and completion of full-length sessions. Questionnaires will be administered with the support of healthcare professionals at each site.

#### Data management and storage

Data will be collected using paper case report forms (CRF), which will include an anonymous ID for each participant. Participants will be provided with the option of completing these CRFs on their own or with the support of the researcher. Data will be input into Qualtrics to generate the dataset, which will be exported into an Excel file and stored securely on password-protected files on University servers at the University of York and King’s College London.

### Data Analysis

To address the primary feasibility outcomes, a variety of approaches will be used. Descriptive statistics will be used to present baseline demographic and clinical data. Categorical data will be presented as frequencies, while continuous data will be presented as means. Summary statistics will be used to describe the proportion of patients approached, who consent to screening, who are eligible, who consent to the study, who are randomised, and who are retained and followed up. The proportion of people who complete the intervention, the number of intervention sessions received, and the number of adverse events will be summarised. A CONSORT flow diagram will be used to present the flow of participants through the study (40). We will provide an overview of the conversion rate from identification, screening, through to consent and randomisation. Completion rates and the proportion of missing data for the clinical outcome measures will also be reported. To account for uncertainty in the sample (i.e. sample error), all estimates will include 95% confidence intervals (CI). We will conduct inferential statistical analysis to explore the time and approach required for analysis in a definitive trial. Analysis of the 3-month clinical outcomes will use mixed-effects models to account for clustering (intra-class correlation). Three-month mean difference in clinical outcomes between the intervention and control arms will be estimated, controlling for the baseline value of the outcome. Since this is a feasibility study, statistical significance testing will not be conducted. Effect sizes (Hedge’s g) and 95% CIs will be estimated and used for interpretation.

## Feasibility Economic Evaluation

A feasibility economic evaluation will be embedded within the feasibility cRCT; therefore, participants will provide consent to take part in this element of the study during recruitment to the feasibility cRCT. As this is a feasibility economic evaluation, we will not calculate any estimates of cost-benefit, effectiveness, or utility. Instead, this part of the study aims to explore the feasibility of data collection for an economic evaluation within a definitive trial.

### Data collection

We will evaluate the feasibility of collecting data related to health and social resource use. Health and social care resource use will be captured using a modified Service Use Questionnaire adapted from previous Service Use logs administered to this population (41, 47). Participants will be asked to complete a retrospective questionnaire to report their healthcare experiences over the past month at baseline and at the 3-month follow-up (47). This will capture information on care received through primary care, outpatient departments, emergency departments, inpatient hospitalisations, and mental health services. The outcome of interest will be the proportion of missing data and the acceptability of using these questionnaires, which will be explored in the qualitative process evaluation. The EQ-5D-5L will be administered to participants at baseline and at three-month follow-up, alongside the clinical outcome questionnaires(48). EQ-5D-5L is used to calculate cost-utility in definitive trials; therefore, we will aim to determine the feasibility of administering the EQ-5D-5L within a definitive trial and the degree to which it contributes to participant burden. The outcome of interest will be the acceptability of the EQ-5D-5L, including completion rates, the proportion of missing data and participants’ experience.

### Data Analysis

Descriptive statistics of the response rates, missing data and costs will be provided(49). This descriptive approach is recommended for feasibility studies, as it provides an overview of the feasibility of the data collection methods without attributing a cause to any differences observed in the data (49). Costs will be estimated according to NHS reference costs and presented as a descriptive cost-consequence analysis. Missing data for the service use questionnaires and the EQ-5D-5L will be presented as frequencies to assess the feasibility of data collection in a future cRCT. The acceptability of using service use questionnaires to collect healthcare use data will also be explored in the qualitative process evaluation.

#### Data management and storage

Data will be collected using digital or paper case report forms (CRF), which will include the anonymous ID for each participant. Participants will have the option to complete these CRFs independently or with the researcher's support. Data will be input into Qualtrics, exported as an Excel file and stored securely on password-protected files on University servers at the University of York and King’s College London.

## Process evaluation

### Participants

Participants will include patients who have participated in the feasibility cRCT, their caregivers, and healthcare workers with experience of the BA intervention. We aim to recruit 15 patients who participated in the study, 10 healthcare workers who either delivered the intervention or had the opportunity to work alongside its delivery, and five carers of individuals who received the integrated BA intervention (36).

Healthcare workers will be eligible for participation in the study if:

- They are a member of a multidisciplinary team, including nurses, medical doctors, healthcare support workers, physiotherapists, and social workers.
- They have had exposure to the intervention, either as a facilitator who delivered it or as a member of the team present in the unit during its delivery.
- They have at least three months of experience within the kidney care setting.

Carers will be eligible for participation in the study if:

- They are a family member or friend who provides support to a participant in the study who received the integrated BA intervention.
- They are over the age of 18.
- They have the capacity to provide informed consent.

We will recruit patients from both the intervention and control groups to explore the acceptability of both the intervention and the trial procedures.

### Recruitment

Patients recruited into the feasibility cRCT will have the opportunity to participate in the qualitative process evaluation. They will be provided with a further participant information sheet providing an overview of the qualitative process evaluation, which will have been developed in collaboration with our PPI panel. Consent will be collected before the commencement of the interview. We will attempt to recruit a diverse range of patient participants across the two sites to ensure that the interviews capture a broad spectrum of experiences across demographics.

Healthcare workers who have delivered the BA intervention or were on shift during its delivery will be approached directly by a researcher to invite them to participate in the process evaluation. They will receive a participant information sheet and will be given at least 24 hours to decide whether they wish to participate.

Carers will be recruited through patients who received the BA intervention as part of the feasibility cRCT. Participants who received the intervention will be provided with a recruitment pack to provide to a family member or friend. All participant-facing materials will be developed in collaboration with our PPI panel.

### Data collection

We aim to conduct 35 semi-structured interviews, comprising 15 with participants who have taken part in the study, 15 with healthcare workers who have experience with the intervention (including intervention facilitators), and five with carers of participants who received the intervention. Semi-structured interviews will enable an in-depth exploration of the experiences within the feasibility cRCT, while also allowing data collection to fit in with the work commitments of healthcare workers and the treatment burden of patients. Interviews will be conducted in a suitable location for participants. In previous feasibility studies set in haemodialysis units, this is frequently during the workday or during haemodialysis treatment(50).

The semi-structured interviews will follow a topic guide structured around the theoretical framework of acceptability (51). They will focus on the acceptability of the integrated BA intervention and the trial procedures (including screening with the PHQ-4). We will explore patients’ experiences during the trial, including any changes to care or life events that may have influenced their ability to engage in the trial. Additionally, we will examine the acceptability of the BA training among healthcare workers and identify any unmet training needs. These interview guides will be informed, developed, and refined through consultation with the PPI panel. The interviews will be audio-recorded and transcribed verbatim.

### Data Analysis

Thematic analysis will be conducted, using NVivo, to identify overarching themes in the data. The interviews will be coded line by line, and these codes will then be reviewed to enable grouping of relevant codes together. These groups will then be iteratively revised to identify the common themes and sub-themes in the data (52). We will present the themes to our PPI panel to explore their perspective on the findings and how the findings can be used to inform a definitive trial and their relevance to practice.

#### Data management and storage

Data will be recorded verbatim using encrypted recording devices. These files will be transferred securely onto University servers (University of York and King’s College London) and will be password-protected. These audio files will be transcribed verbatim. Any transferring of the data will be done using secure university drop-off services. During transcription, the interviews will be pseudonymised, with all identifying information removed. Audio recordings will be deleted once the data has been transcribed and checked for accuracy.

## Progression Criteria

Progression to a definitive cRCT will be determined by recruitment rates and the acceptability of the methods and intervention for patients and staff. Both quantitative and qualitative data will be used to determine the feasibility of a cRCT (41, 53):

- Recruitment
  - 75-100% of the target sample size recruited across the two sites will result in progression to a pilot study and definitive trial.
  - 50-74% of the target sample size recruited across the two sites will result in progression to a pilot study, following appropriate amendments to address barriers to recruitment.
  - 25-50% of the target sample size recruited across the two sites will result in progression to a pilot study following review by the study steering committee and future co-applicants to ensure that adequate revisions are made to address barriers to recruitment.
  - <25% of the target sample size recruited will result in the study not progressing to a full trial.
- Randomisation
  - Evidence of substantial or consistent contamination from the method of clustering (either due to high rates of contamination or evidence of significant contamination from qualitative interviews) will result in a review of the randomisation and clustering method, before progression to a definitive trial.
- Intervention
  - Progression to a full trial will be contingent on the acceptability of the integrated BA intervention for both patients and healthcare professionals. This will be determined by exploration within the qualitative process evaluation. Any necessary modifications or adaptations will be made in collaboration with our PPI panel, before progression to a definitive trial.
- Data collection
  - The use of the outcome measures in a future trial will depend on their acceptability, determined by the proportion of missing information, and through the qualitative process evaluation.

## Definition of end of study

End of study will be defined as the date at which the last participant has completed their final study process, defined as:

- Completion of the final planned follow-up assessment in the study (3 months)
- Completion of the final planned interview in the study
- Full withdrawal from follow-up for any reason

## Ethical considerations

### Informed consent

To ensure we obtain informed consent, potentially eligible patients will be identified by clinical gatekeepers within the dialysis unit, ensuring that patients are only screened if they have the capacity to consent. Participants will be provided with a participant information sheet that provides an overview of the study, drafted in collaboration with the PPI panel to ensure the language is accessible and easy to understand. Potential participants will also be provided with the opportunity to ask the researcher questions about their participation in the study. Contact details will also be provided for the researcher and lead investigators in case the potential participant has any further questions. Once identified as eligible, potential participants will be given 48 hours to consider their participation in the study before they are provided with a consent form.

A similar process will be followed to obtain informed consent for the qualitative process evaluation. At post-intervention data collection, the researcher will provide participants with an overview of the qualitative process evaluation and a participant information sheet. The researcher will give the participant at least 48 hours to consider whether they would like to participate in the interviews before approaching them to arrange the time and place of the interview and obtain written informed consent.

Healthcare professionals, including the intervention facilitators and other staff who were present on the unit during the intervention's delivery, will be approached by the researcher and provided with their own participant information sheet regarding the qualitative process evaluation. They will be given the opportunity to ask questions and provided at least 24 hours to consider participation before the researcher arranges the time and place of the interview and obtains written informed consent.

### Confidentiality and anonymity

Participants will be assigned anonymous codes upon enrolment in the study. Data will be collected and stored in accordance with the EU General Data Protection Regulation. All identifiable information will be confidential, hard copies of personal data will be stored in a locked filing cabinet, in a locked room in a building with restricted access in the University of York and King’s College London. Electronic data will be stored in password-protected, encrypted files on secure university servers at the University of York or King’s College London. Data will be stored for a minimum of 10 years and subsequently destroyed. For qualitative analysis, all interview participants will be provided with an anonymous code, and any identifying information will be removed from the verbatim transcripts. Participants will be informed of the limits of confidentiality and anonymity prior to obtaining informed consent, and this information will also be included on the participant information sheets.

During the development of the BA intervention, renal nurses raised concerns about maintaining confidentiality during BA intervention sessions, as haemodialysis units are typically open-plan with several people in the same bay. This was raised and discussed with PPI panel members, who advised that it was typical for personal, sometimes sensitive, issues to be discussed while they were on haemodialysis treatment, that most people were not listening in to others' consultations, and that they didn’t view the intervention sessions as something uniquely different to this usual practice. While the intervention sessions do not focus on highly sensitive subjects, such as past trauma, there is still a chance that participants may feel uncomfortable discussing symptoms of depression or maladaptive behaviours in the context of an open dialysis unit. Therefore, we have included a flexible approach to these subjects in the intervention training, allowing participants to work through these sections independently, prior to dialysis, or actively with the facilitator at the bedside.

### Welfare of participants

This research will involve people with an advanced illness who are receiving a life-sustaining treatment in a highly specialised clinical area. Research staff will be instructed to maintain infection control standards and not impede the work of clinical staff at the unit. Intervention facilitators will be renal healthcare professionals who have experience providing support to people receiving haemodialysis, and therefore will be familiar with the clinical setting and patient population.

The focus of the research is on depression, and we will use the PHQ-4 to screen participants for eligibility and the PHQ-9 as a clinical outcome measure. The PHQ-9 includes an item which asks respondents whether they have experienced ‘Thoughts that you would be better off dead, or thoughts of hurting yourself in some way?’. A positive answer to this question (i.e. any answer other than ‘not at all’) could suggest risk of self-harm or suicide. We have developed a suicide risk protocol that will be triggered if participants provide a positive answer to this item, or if they express thoughts of self-harm, suicide or life not worth living during the intervention sessions.

There is also a risk that some patients will consent to screening and screen as high risk for depression, but decline participation in the study. We will provide all patients who are screened and score over the threshold for participation with information about available support services and how they can access mental health support, regardless of whether they consent to participate.

There is a risk that participants will become distressed during the intervention sessions, as they may discuss symptoms of depression or the impact that haemodialysis has had on their usual activities. Although the focus is not on highly distressing subject matter, there is also a risk that participants may become distressed during the qualitative interviews as part of the process evaluation. Therefore, we have developed a series of distress protocols to ensure research staff and intervention facilitators can respond appropriately if this occurs.

### Welfare of the research staff and facilitators

All intervention facilitators will be trained to deliver the BA intervention and will receive ongoing support from the broader research team during its implementation. This will include supervision from the project co-lead, Professor Lina Gega. This will provide facilitators with an opportunity to raise concerns and discuss any difficulties they have encountered during delivery.

As the study focuses on a population with an advanced illness who are experiencing depression, there is a risk that recruitment or data collection may have an emotional impact on research staff. Therefore, research staff will be encouraged to debrief with the broader research team if they encounter any issues during recruitment or data collection.

## References

1.	UK KR. What is haemodialysis? 2024 [Available from: <https://www.kidneyresearchuk.org/kidney-health-information/living-with-kidney-disease/care-and-treatments-for-people-with-kidney-failure/haemodialysis/>.

2.	Palmer S, Vecchio M, Craig JC, Tonelli M, Johnson DW, Nicolucci A, et al. Prevalence of depression in chronic kidney disease: systematic review and meta-analysis of observational studies. Kidney Int. 2013;84(1):179–91.

3.	Arias de la Torre J, Vilagut G, Ronaldson A, Dregan A, Ricci-Cabello I, Hatch SL, et al. Prevalence and age patterns of depression in the United Kingdom. A population-based study. J Affect Disord. 2021;279:164–72.

4.	Gebrie MH, Ford J. Depressive symptoms and dietary non-adherence among end stage renal disease patients undergoing hemodialysis therapy: systematic review. BMC Nephrology. 2019;20(1):429.

5.	Wu PH, Lin MY, Huang TH, Lin YT, Hung CC, Yeh YC, et al. Depression amongst patients commencing maintenance dialysis is associated with increased risk of death and severe infections: A nationwide cohort study. PLoS One. 2019;14(6):e0218335.

6.	UK KC. Psychosocial health - a manifesto for action. Kidney Care UK; 2022.

7.	Chilcot J, Pearce CJ, Hall N, Busby AD, Hawkins J, Vraitch B, et al. The identification and management of depression in UK Kidney Care: Results from the Mood Maps Study. J Ren Care. 2024;50(3):297–306.

8.	Chilcot J, Hudson JL. Is successful treatment of depression in dialysis patients an achievable goal? Semin Dial. 2019;32(3):210–4.

9.	Friedli K, Guirguis A, Almond M, Day C, Chilcot J, Da Silva-Gane M, et al. Sertraline Versus Placebo in Patients with Major Depressive Disorder Undergoing Hemodialysis: A Randomized, Controlled Feasibility Trial. Clin J Am Soc Nephrol. 2017;12(2):280–6.

10.	Pena-Polanco JE, Mor MK, Tohme FA, Fine MJ, Palevsky PM, Weisbord SD. Acceptance of Antidepressant Treatment by Patients on Hemodialysis and Their Renal Providers. Clin J Am Soc Nephrol. 2017;12(2):298–303.

11.	Pearce CJ, Hall N, Hudson JL, Farrington K, Tucker MJR, Wellsted D, et al. Approaches to the identification and management of depression in people living with chronic kidney disease: A scoping review of 860 papers. J Ren Care. 2024;50(1):4–14.

12.	Duarte PS, Miyazaki MC, Blay SL, Sesso R. Cognitive-behavioral group therapy is an effective treatment for major depression in hemodialysis patients. Kidney International. 2009;76(4):414–21.

13.	Hudson JL, Moss-Morris R, Game D, Carroll A, Chilcot J. Improving Distress in Dialysis (iDiD): A tailored CBT self-management treatment for patients undergoing dialysis. J Ren Care. 2016;42(4):223–38.

14.	Uphoff E, Pires M, Barbui C, Barua D, Churchill R, Cristofalo D, et al. Behavioural activation therapy for depression in adults with non-communicable diseases. Cochrane Database Syst Rev. 2020;8(8):Cd013461.

15.	Cuijpers P, van Straten A, Warmerdam L. Behavioral activation treatments of depression: a meta-analysis. Clin Psychol Rev. 2007;27(3):318–26.

16.	Richards DA, Ekers D, McMillan D, Taylor RS, Byford S, Warren FC, et al. Cost and Outcome of Behavioural Activation versus Cognitive Behavioural Therapy for Depression (COBRA): a randomised, controlled, non-inferiority trial. Lancet. 2016;388(10047):871–80.

17.	Ekers D, Webster L, Van Straten A, Cuijpers P, Richards D, Gilbody S. Behavioural Activation for Depression; An Update of Meta-Analysis of Effectiveness and Sub Group Analysis. PLOS ONE. 2014;9(6):e100100.

18.	(NICE) NIfHaCE. Depression in adults with a chronic physical health problem: recognition and

management [Clinical guideline [CG91]. In: (NICE) NIfHaCE, editor. 2009.

19.	Carswell C, Brown JVE, Lister J, Ajjan RA, Alderson SL, Balogun-Katung A, et al. The lived experience of severe mental illness and long-term conditions: a qualitative exploration of service user, carer, and healthcare professional perspectives on self-managing co-existing mental and physical conditions. BMC Psychiatry. 2022;22(1):479.

20.	Cogley C, Carswell C, Bramham K, Chilcot J. Chronic Kidney Disease and Severe Mental Illness: Addressing Disparities in Access to Health Care and Health Outcomes. Clinical Journal of the American Society of Nephrology. 2022;17(9).

21.	Carswell C, Cogley C, Bramham K, Chilcot J, Noble H, Siddiqi N. Chronic kidney disease and severe mental illness: a scoping review. Journal of Nephrology. 2023;36(6):1519–47.

22.	Roberti J, Cummings A, Myall M, Harvey J, Lippiett K, Hunt K, et al. Work of being an adult patient with chronic kidney disease: a systematic review of qualitative studies. BMJ Open. 2018;8(9):e023507.

23.	Moran A, Scott PA, Darbyshire P. Existential boredom: the experience of living on haemodialysis therapy. Med Humanit. 2009;35(2):70–5.

24.	Raue PJ, Hawrilenko M, Corey M, Lin J, Chen S, Mosser BA. “Do More, Feel Better”: Pilot RCT of Lay-Delivered Behavioral Activation for Depressed Senior Center Clients. Behavior Therapy. 2022;53(3):458–68.

25.	Chen GJ, Kunik ME, Marti CN, Choi NG. Cost-effectiveness of Tele-delivered behavioral activation by Lay counselors for homebound older adults with depression. BMC Psychiatry. 2022;22(1):648.

26.	Magidson JF, Lejuez CW, Kamal T, Blevins EJ, Murray LK, Bass JK, et al. Adaptation of community health worker-delivered behavioral activation for torture survivors in Kurdistan, Iraq. Glob Ment Health (Camb). 2015;2.

27.	Ekers D, Godfrey C, Gilbody S, Parrott S, Richards DA, Hammond D, et al. Cost utility of behavioural activation delivered by the non-specialist. British Journal of Psychiatry. 2011;199(6):510–1.

28.	Hudson JL, Moss-Morris R, Norton S, Picariello F, Game D, Carroll A, et al. Tailored online cognitive behavioural therapy with or without therapist support calls to target psychological distress in adults receiving haemodialysis: A feasibility randomised controlled trial. J Psychosom Res. 2017;102:61–70.

29.	Craig P, Dieppe P, Macintyre S, Michie S, Nazareth I, Petticrew M. Developing and evaluating complex interventions: the new Medical Research Council guidance. BMJ. 2008;337:a1655.

30.	Skivington K, Matthews L, Simpson SA, Craig P, Baird J, Blazeby JM, et al. A new framework for developing and evaluating complex interventions: update of Medical Research Council guidance. BMJ. 2021;374:n2061.

31.	Anderson BM, Qasim M, Correa G, Evison F, Gallier S, Ferro CJ, et al. Depression is associated with frailty and lower quality of life in haemodialysis recipients, but not with mortality or hospitalization. Clin Kidney J. 2023;16(2):342–54.

32.	Picariello F, Hulme K, Seaton N, Hudson JL, Norton S, Wroe A, et al. A randomized controlled trial of a digital cognitive-behavioral therapy program (COMPASS) for managing depression and anxiety related to living with a long-term physical health condition. Psychological Medicine. 2024;54(8):1796–809.

33.	Sim J. Should treatment effects be estimated in pilot and feasibility studies? Pilot Feasibility Stud. 2019;5:107.

34.	Arain M, Campbell MJ, Cooper CL, Lancaster GA. What is a pilot or feasibility study? A review of current practice and editorial policy. BMC Medical Research Methodology. 2010;10(1):67.

35.	Carswell C, Reid J, Walsh I, Johnston W, McAneney H, Mullan R, et al. A mixed-methods feasibility study of an arts-based intervention for patients receiving maintenance haemodialysis. BMC Nephrology. 2020;21(1):497.

36.	Guest G, Bunce A, Johnson L. How Many Interviews Are Enough?: An Experiment with Data Saturation and Variability. Field Methods. 2006;18(1):59–82.

37.	Tindall L, Gega, L. Community-Based Behavioural Activation Training (ComBAT) for Depression: Randomised Controlled Trial: ISCTRN Registry; 2022 [Available from: <https://www.isrctn.com/ISRCTN57377955>.

38.	FutureLearn. Introduction to Behavioural Activation for Depression: University of York; 2024 [Available from: <https://www.futurelearn.com/courses/introduction-to-behavioural-activation-for-depression>.

39.	Ahmed N, Zavala GA, Siddiqui F, Aslam F, Keding A, Halmkan S, et al. A randomised controlled feasibility trial of Behavioural activation as a treatment for people with diabetes and depression: (DiaDeM feasibility trial). Journal of Affective Disorders. 2025;372:333–46.

40.	Eldridge SM, Chan CL, Campbell MJ, Bond CM, Hopewell S, Thabane L, et al. CONSORT 2010 statement: extension to randomised pilot and feasibility trials. BMJ. 2016;355:i5239.

41.	Carswell C, Reid J, Walsh I, McAneney H, Noble H. Implementing an arts-based intervention for patients with end-stage kidney disease whilst receiving haemodialysis: a feasibility study protocol. Pilot and Feasibility Studies. 2019;5(1):1.

42.	Eldridge SM, Lancaster GA, Campbell MJ, Thabane L, Hopewell S, Coleman CL, et al. Defining Feasibility and Pilot Studies in Preparation for Randomised Controlled Trials: Development of a Conceptual Framework. PLOS ONE. 2016;11(3):e0150205.

43.	Watnick S, Wang PL, Demadura T, Ganzini L. Validation of 2 depression screening tools in dialysis patients. Am J Kidney Dis. 2005;46(5):919–24.

44.	Spitzer RL, Kroenke K, Williams JB, Löwe B. A brief measure for assessing generalized anxiety disorder: the GAD-7. Arch Intern Med. 2006;166(10):1092–7.

45.	Peipert JD, Bentler PM, Klicko K, Hays RD. Psychometric Properties of the Kidney Disease Quality of Life 36-Item Short-Form Survey (KDQOL-36) in the United States. Am J Kidney Dis. 2018;71(4):461–8.

46.	Tennant R, Hiller L, Fishwick R, Platt S, Joseph S, Weich S, et al. The Warwick-Edinburgh Mental Well-being Scale (WEMWBS): development and UK validation. Health Qual Life Outcomes. 2007;5:63.

47.	Leggett LE, Khadaroo RG, Holroyd-Leduc J, Lorenzetti DL, Hanson H, Wagg A, et al. Measuring Resource Utilization: A Systematic Review of Validated Self-Reported Questionnaires. Medicine (Baltimore). 2016;95(10):e2759.

48.	Zhou T, Guan H, Wang L, Zhang Y, Rui M, Ma A. Health-Related Quality of Life in Patients With Different Diseases Measured With the EQ-5D-5L: A Systematic Review. Front Public Health. 2021;9:675523.

49.	Apantaku G, Mitton C, Wong H, Ho K. Home Telemonitoring Technology for Patients With Heart Failure: Cost-Consequence Analysis of a Pilot Study. JMIR Form Res. 2022;6(6):e32147.

50.	Carswell C, Reid J, Walsh I, McKeaveney C, Noble H. The Experiences of a Complex Arts-Based Intervention for Patients with End-Stage Kidney Disease Whilst Receiving Haemodialysis: A Qualitative Process Evaluation. Healthcare (Basel). 2021;9(10).

51.	Sekhon M, Cartwright M, Francis JJ. Acceptability of healthcare interventions: an overview of reviews and development of a theoretical framework. BMC Health Services Research. 2017;17(1):88.

52.	Braun V, Clarke V. Using thematic analysis in psychology. Qualitative Research in Psychology. 2006;3(2):77–101.

53.	Brown JVE, Ajjan R, Alderson S, Böhnke JR, Carswell C, Doherty P, et al. The DIAMONDS intervention to support self-management of type 2 diabetes in people with severe mental illness: Study protocol for a single-group feasibility study. SSM - Mental Health. 2022;2:100086.
